# Studies of a Murine Monoclonal Antibody Directed against DARC: Reappraisal of Its Specificity

**DOI:** 10.1371/journal.pone.0116472

**Published:** 2015-02-23

**Authors:** Dorota Smolarek, Claude Hattab, Anna Buczkowska, Radoslaw Kaczmarek, Anna Jarząb, Sylvie Cochet, Alexandre G. de Brevern, Jolanta Lukasiewicz, Wojciech Jachymek, Tomasz Niedziela, Magdalena Grodecka, Kazimiera Wasniowska, Yves Colin Aronovicz, Olivier Bertrand, Marcin Czerwinski

**Affiliations:** 1 Laboratory of Glycoconjugate Immunochemistry Ludwik Hirszfeld Institute of Immunology and Experimental Therapy, Wrocław, Poland; 2 Laboratory of Microbial Immunochemistry and Vaccines Ludwik Hirszfeld Institute of Immunology and Experimental Therapy, Wrocław, Poland; 3 Laboratory of Medical Microbiology, Ludwik Hirszfeld Institute of Immunology and Experimental Therapy, Wrocław, Poland; 4 INSERM, UMR_S1134, F-75015 Paris, France; 5 Institut National de la Transfusion Sanguine, F-75015 Paris, France; 6 Institute of Biochemistry and Biophysics, Warsaw, Poland; 7 Institute of Physiotherapy, Faculty of Physical Education and Physiotherapy, Opole University of Technology, Opole, Poland; 8 Universite Paris-Diderot, Sorbonne Paris Cité, F-15013, Paris, France; 9 Laboratory of Excellence GR-Ex, Paris, France; 10 Division of Transplantation Immunology and Mucosal Biology, MRC Centre for Transplantation, King’s College London, Guy’s Hospital, London, United Kingdom; Centro de Pesquisa Rene Rachou/Fundação Oswaldo Cruz (Fiocruz-Minas), BRAZIL

## Abstract

Duffy Antigen Receptor for Chemokines (DARC) plays multiple roles in human health as a blood group antigen, a receptor for chemokines and the only known receptor for *Plasmodium vivax* merozoites. It is the target of the murine anti-Fy6 monoclonal antibody 2C3 which binds to the first extracellular domain (ECD1), but exact nature of the recognized epitope was a subject of contradictory reports. Here, using a set of complex experiments which include expression of DARC with amino acid substitutions within the Fy6 epitope in *E. coli* and K562 cells, ELISA, surface plasmon resonance (SPR) and flow cytometry, we have resolved discrepancies between previously published reports and show that the basic epitope recognized by 2C3 antibody is ^22^FEDVW^26^, with ^22^F and ^26^W being the most important residues. In addition, we demonstrated that ^30^Y plays an auxiliary role in binding, particularly when the residue is sulfated. The STD-NMR studies performed using 2C3-derived Fab and synthetic peptide corroborated most of these results, and together with the molecular modelling suggested that ^25^V is not involved in direct interactions with the antibody, but determines folding of the epitope backbone.

## Introduction

Duffy Antigen Receptor for Chemokines (DARC) is a seven-transmembrane domain glycoprotein present on red blood cells (RBC) and post-capillary endothelial cells or Purkinje cells. DARC plays an important role in human health, being a blood group antigen, the only known receptor for *Plasmodium vivax* parasite that causes malaria and a receptor for chemokines.

DARC glycoprotein carries blood group antigens Fy^a^ and Fy^b^, which are encoded by two allelic genes, designated FY*01 and FY*02 respectively. The Fy^a^/Fy^b^ polymorphism depends on the presence of Gly or Asp residue at position 42 in the polypeptide chain of DARC (Gly in Fy^a^, Asp in Fy^b^). The third common allele called FY*02N.01 occurs mostly in Africans or African-American people and results in the lack of DARC expression on erythrocytes, but not on other tissues. That phenotype, called Fy(a-b-) is caused by a single nucleotide polymorphism at position -67T>C in the GATA box of DARC gene promoter [1] and it was thought for decades that it was associated with resistance of red blood cells to infection by *Plasmodium vivax* merozoites [2]. However, recent data cast some doubt on this dogma, suggesting that another receptor for *P. vivax* may exist [3]. *P. vivax* is the most widespread species of parasite causing malaria with about 2.85 billion of people living in endemic regions. DARC is recognized by *P. vivax* Duffy Binding Protein (PvDBP), and thus serves as a receptor for *P. vivax* merozoites [4, 5]. The crystal structure of DARC-PvDBP complex has been recently resolved [5].

DARC is also a chemokine receptor belonging to G-Protein Coupled Receptor (GPCR) family [6, 7], and this function most probably determines DARC’s involvement in malignancies [8–10], inflammation [11], HIV infection or AIDS progression [12–14]. DARC binds chemokines [15], but lacks the DRY motif which is typical for this family of receptors and required for signal transduction, hence it was considered a “silent chemokine receptor” [16, 17]. Recently, it was proposed to call it Atypical Chemokine Receptor 1 (ACKR1) [18]. It was shown that DARC plays an important role in regulation of the chemokine level, but in a different manner depending on its localization: on the erythrocytes it serves as a reservoir of chemokines, while on the endothelial cells it facilitates chemokines translocation through the vessel walls.

DARC contains three N-linked oligosaccharide chains located at Asn-16, 27, and 33 [19]. These moieties are mostly of triantennary complex type, terminated with sialic acid residues [19–21]. In addition, Tyr-30 and 41 are sulfated [22], and sulfation of Tyr-41 increases binding of PvDBP to DARC approximately thousand times [22].

There are several epitopes recognized by human and murine antibodies on DARC: two of them determine the Fy^a^/Fy^b^ blood group status, the other is Fy3, a conformational epitope consisting of fragments of extracellular domains 1 and 3, and a linear epitope called Fy6, present on the ECD1 of the protein.

Specificities of several monoclonal anti-Fy6 antibodies have been precisely defined [23–25]. It was found that all tested antibodies with anti-Fy6 specificity recognize linear epitopes containing ^19^QLDFEDV^25^ sequence located between two potential N-glycosylation sites of Duffy protein, Asn-16 and Asn-27. One of the widely used anti-DARC antibodies, monoclonal antibody 2C3 with anti-Fy6 specificity, was obtained after immunization of mice with CHO cells transfected with the vector encoding DARC. Its epitope was evaluated by Wasniowska et al. using Pepscan analysis, and determined to be a linear sequence ^22^FEDVW^26^, in which only Asp-24 may be replaced by other amino acids without influencing binding of the antibody [24]. In contrast, Tournamille et al., using recombinant forms of DARC expressed in K562 cells suggested that Trp-26 can be substituted by other amino acids without losing reactivity to 2C3 antibody. In addition, it was claimed that the antibody does not bind to the recombinant form of Duffy antigen, in which Tyr-30 is substituted by another amino acid [25].

Since antibodies that recognize Fy6 epitope can inhibit interaction between chemokines [26] or PvDBP [27] and DARC, the exact determination of epitope recognized by 2C3 antibody is of great importance. The aim of our study was to precisely characterize the epitope recognized by 2C3 antibody and to resolve discrepancies between the two abovementioned reports. To this end, we prepared two sets of constructs: one set comprised the full-length consensus DARC or mutants with substitutions introduced in the first extracellular domain (ECD1); these constructs were expressed in K562 cells. The other set comprised the consensus or mutant ECD1 (with the same substitutions as in the full-length DARC mutants) fused in-frame to nuclease from *Staphylococcus aureus* and expressed in *E. coli*. The proteins obtained in *E. coli* or K562 cells were used to evaluate the binding of 2C3 antibody using ELISA, SPR and flow cytometry. In addition, we employed saturation transfer difference spectroscopy (STD-NMR) of a 2C3-derived Fab fragment with a synthetic peptide encompassing the Fy6 epitope in order to precisely define how the side chains of amino acids interact with the Fab.

## Materials and Methods

### Monoclonal antibody

MAb 2C3 (clone name NaM185-2C3) was obtained previously by immunizing mice with CHO cells transfected with the vector encoding DARC protein [24].

### Recombinant proteins used as reagents for 2C3 MAb studies

Constructs with substitutions of amino acids located within or in the vicinity of the Fy6 epitope were prepared by mutagenesis using plasmid containing first extracellular domain of DARC followed by nuclease from *Staphylococcus aureus* as a template [26].

The protein (called thereafter ECD1-nuc) was expressed in *E. coli* BL21 strain (New England Biolabs, Ipswich, MA, USA) grown in autoinduction medium, and purified from whole cell extract using ion exchange Capto-S column (GE Healthcare, Little Chalfont, UK) followed by reversed-phase liquid chromatography on a Vydac TCP 104 column (Grace, Columbia, MD, USA).

The full-length DARC was purified from K562 cells expressing DARC, essentially as described [26] by immunochromatography on an immobilized camel antibody fragment (nanobody), but using a column format. Briefly, 16 × 10^8^ cells were lyzed in 300 ml of lysis buffer (20 mM Tris-HCl pH 8.0 containing 0.15 M NaCl, 5 mM EDTA, 1% Triton X100 and protease inhibitors (cOmplete, Roche, Basel, Switzerland). After centrifugation, supernatant was loaded at 15 ml/hour onto a 1 ml affinity column (prepared using NHS-activated Sepharose and purified anti-DARC nanobody). After washing the column with lysis buffer followed by 20 mM Tris-HCl pH 8.0 containing 0.15 M NaCl and 0.3% C12E8 detergent (Sigma), DARC was eluted by passing onto the colum 5 ml of peptide DFEDVW (dissolved in the latter buffer at 4 mg/ml concentration). Column was further rinsed with 0.1 M Glycine pH 2.8. DARC was freed of peptide by dialysis. The DARC purity was evaluated by SDS-PAGE (S1 Fig.).

### Cloning and expression of 2C3 Fab fragment

cDNA encoding variable regions of the 2C3 Fab fragment was cloned by PCR using total RNA extracted from 2C3-producing hybridoma cells. The amplified DNA was cloned into pComb3H vector, expressed in *E. coli* and purified as described previously [28, 29]. The Fab purity was evaluated by SDS-PAGE (S2 Fig.).

### ELISA

Microtiter 96-well plate (MaxiSorb, Nunc, Roskilde, Denmark) was coated with wild-type ECD1-nuc 0.1 μg of protein per well in 50 μl of carbonate buffer pH 9.6, overnight at 4°C. Nonspecific binding was blocked by incubation for 1 hour at room temperature with TBS (Tris-buffered saline: 20 mM Tris-HCl, and 150 mM NaCl pH 7.4) containing 5% non-fat dry milk. After washing the wells once with TBS, 50 μl of 2C3 MAb or 2C3 Fab fragments appropriately diluted in TBS were added to each well, and the mixture was incubated for 1 h at room temperature. After five washes with TBS, 50 μl of a 1000 x dilution in TBS of rabbit anti-Fab antiserum conjugated with alkaline phosphatase or avidin-alkaline phosphatase 1000 x diluted (Pierce, Rockford, IL, USA) were added to each well and the plates were incubated for 1 h at room temperature. To develop the reaction, p-nitrophenylphosphate was used (1 mg/ml in 60 mM carbonate buffer, pH 9.5, containing 1 mM MgCl_2_); the OD_405_ was determined using Perkin Elmer EnSpire 2300 Multilabel Reader (Waltham, MA, USA). All experiments were performed in triplicates.

Inhibition of the antibody binding was evaluated by incubating appropriately diluted MAb or Fab with serially diluted ECD1-nuc mutants for 1h. The samples were then applied on the coated plate and incubated for 10 min, and the bound antibody was detected as described above. The percentage of inhibition was calculated as: [1- (OD_405_ of sample)/(OD_405_ of control)] x100.

### Surface plasmon resonance (SPR)

All experiments were performed in the Biacore X100 machine using CM5 type sensor chips (GE Healthcare), the running buffer was HBS-EP (GE Healthcare). To immobilize proteins, amine coupling procedure was used according to the manufacturer’s instructions. 2C3 MAb or 2C3 Fab was immobilized in the flow cell, and murine monoclonal anti-Kx2 antibody [30] was immobilized in the reference cell. Approximately 400 RU of protein was immobilized in each flow cell. ECD1-nuc with wild type or mutated sequences were used as analytes, and each construct was evaluated in 5 different concentrations. 50 mM HCl was used for regeneration of the chip. The association phase (binding of ligand) was run for 100 sec., and then dissociation was continued for 600 sec. The results were processed using BIAevaluation software and 1:1 Langmuir binding model.

For SPR inhibition assay, 2000 RU of WT ECD1-nuc construct was immobilized on Fc2 channel of a sensor chip as described above, with *S. aureus* nuclease in the Fc1 reference cell. 2C3 MAb (culture supernatant diluted 40 times) was incubated with the ECD1-nuc constructs at room temperature for one hour, the samples were then applied to the chip, the association phase was continued for 100 sec., and the RU value was read at 90 sec. The obtained values were used to calculate the concentration of each ECD1 mutant causing 50% inhibition of binding of 2C3 to ECD1-nuc. The synthetic peptides with sequences DFEDVWNSSYG and DFEDVWNSSY(SO_3_H)G were obtained from Cambridge Research Biochemicals, Clevelend, UK.

To evaluate how the presence of a negative charge on tyrosine residues influences the antigen binding, the ECD1-nuc construct was phosphorylated by Src kinase (Cell Signalling Technology, Danvers, MA, USA) and binding of 2C3 Fab to the phosphorylated ligand was studied. Phosphorylation of proteins already immobilized on the sensor chip was performed in the Biacore apparatus. Constructs of ECD1-nuc were used as a ligand and nuclease was immobilized in the reference cell (the phosphorylation site on Tyr-54 of nuclease was removed by mutagenesis from both ECD1-nuc and nuclease constructs). The phosphorylation reaction mixture was prepared by mixing Src Kinase (25 ng/μl final concentration), calcium-calmodulin (Sigma, 58 ng/μl final concentration), and ATP (73 μM final concentration) in the buffer supplied by the enzyme manufacturer. The mixture was injected at 5 μl/minute in two pulses of 1060 and 470 seconds onto the chip equilibrated at 37°C.

DARC purified from DARC-expressing K562 cells by immunochromatography on immobilized anti-DARC VHH (S1 Fig.) was immobilized at ca. 400 RU on the analysis flow cell, the reference cell was only activated and deactivated.

Analysis of the influence of glycosylation on binding to DARC was performed by *in situ* deglycosylation of purified DARC immobilized on the chip. Reaction mixture prepared by diluting N-glycosidase (New England Biolabs, Ipswich, MA, USA) to 50 UI/μl in the buffer recommended by the manufacturer was injected three times for 1000 sec. each time, the temperature was set to 37°C and flow rate to 5 μl/min. To evaluate efficiency of the deglycosylation process, *Sambucus nigra* lectin (Vector Labs, Burlingname, CA, USA) in concentration 70 nM and 7 nM was injected.

### Transfection of K562 cells

The mutants were prepared using the Quickchange kit according to the manufacturer’s instruction (Stratagene, La Jolla, CA, USA). 2 × 10^6^ of K562 cells (obtained from ATCC, Manassas, VA, USA, clone number CCL-243) were transfected with vector encoding DARC using Amaxa Nucleofector system (Lonza, Basel, Switzerland). The DARC-expressing cells were selected for resistance to geneticin, as described [19].

### Flow Cytometry analysis

Approximately 10^6^ of K562 cells transfected with vector encoding DARC were used in each analysis. After washing with PBS, the cells were incubated for 1 hour with either one of three antibody solutions in PBS-0.2% BSA: i) anti-mouse IgG (Sigma, St. Louis, MO, USA) as a negative control, ii) anti-Fy^a^ human antibody (clone 665, kindly provided by Dr. F. Buffiere, Bordeaux, France) as a positive control [25], iii) 2C3 MAb culture supernatant. The cells were then washed 3 times with PBS, and incubated in the dark with secondary antibody conjugated to PE (anti-mouse or anti-human, Becton-Dickinson, Franklin Lakes, NJ, USA) for 40 min. After three washes the cells were resuspended in 300 μl of PBS and evaluated by flow cytometry (FaxCalibur, Becton-Dickinson).

### NMR spectroscopy

The NMR spectra of the synthetic peptide (DFEDVWNSSYG) were obtained for ^2^H_2_O solutions at 25°C with a Bruker AVANCE III 600 MHz spectrometer (Bremen, Germany), using acetone (δ_H_ 2.225 and δ_C_ 31.05) as the internal reference. The main groups of signals were assigned by one- and two-dimensional experiments (COSY, TOCSY, ROESY, HMBC, and HSQC-DEPT). In the TOCSY experiments the mixing times were 30, 60, and 100 ms. The delay in the HMBC experiments and the mixing time in the ROESY experiments were 60 and 200 ms, respectively. The data were acquired and processed using standard Bruker software. The processed spectra were assigned with help of the SPARKY program [31].

One-dimensional STD NMR experiments were performed at 25°C with a 5-mm inverse-detection QCI cryoprobe equipped with z-gradient. Spectra were recorded using the pulse programs bundled with the Topspin software. The excitation sculpting pulse sequences were used to suppress water signals in the spectra. The broad resonances of a protein were suppressed with a 20-ms spin-lock pulse. The protein was irradiated at δ_H_ −0.5 ppm and 9.5 ppm (on-resonance) and δ_H_30 ppm (off-resonance) with a train of Gaussian shaped pulses (50 ms) and the data were collected as a pseudo2D, split into 1D on-resonance and off-resonance spectra and followed by calculation of the difference spectra.

The setup of the STD NMR experiments was optimized in a series of experiments with ligand-protein and ligand-only samples to ensure that the irradiation at the selected frequency did not affect the ligand. The saturation time used in the experiments was 2 s. Samples (total volume, 160 μl) were prepared in 3 mm NMR tubes, using PBS made with ^2^H_2_O, pH 7.5 (not corrected for ^2^H_2_O). Fab/peptide ratios of 1:46 (21 μM Fab with ~1 mM peptide) were used.

### Analysis of Peptides Structure and Folding

The secondary structure and folding of DFED**V**WNSSYG and peptides with Val-25 substitutions were predicted and calculated by PEP-FOLD, an online resource for *de novo* peptide structure prediction in aqueous solutions [32]. The PEP-FOLD provides pdb (Protein Data Bank) files with representation of different peptide models combined in clusters. The models obtained by molecular modeling of peptides with the lowest sOPEP energy (sOPEP—Optimized Potential for Efficient Structure Prediction) were used for further analysis. The pdb files were visualized and analyzed by Chimera software and the distances between Trp-26, Tyr-30 and Phe-22, residues involved in the peptide-antibody interaction were calculated [33].

## Results

### Evaluation of the epitope recognized by 2C3 monoclonal antibody

Soluble ECD1-nuc constructs with consensus-type sequence or the sequence containing amino acid substitutions were tested by ELISA for ability to inhibit binding of the antibody to ECD1-nuc immobilized on a polystyrene plate. Profiles of inhibition of 2C3 MAb by different ECD1-nuc constructs are shown in S3 Fig. The binding strength of a given construct to antibody was calculated as the concentration of protein required for 50% inhibition of binding to the immobilized wild type construct (Table 1). The results demonstrate that only constructs in which Trp-26 is present in the epitope can inhibit, at low concentration, binding of the 2C3 antibody to coated ECD1-Nuc (Table 1, compare rows 1-3 with rows 4-8). Substitution of this residue by another aromatic amino acid or Ala results in a decrease of inhibition (Table 1, rows 6-8). However, this effect was somewhat compensated for when a negative charge was introduced at position 30 by replacing Tyr with Glu (Table 1, rows 4 and 5).

**Table 1:** Inhibition of binding of 2C3 MAb to ECD1-nuc constructs evaluated by ELISA.

**No.**	**Sequence**	**Concentration required for 50% inhibition [μg/ml]**
1	FEDV**W**NSS**Y**	0.17±0.19
2	FEDV**W**NSS**E**	0.22±0.12
3	FEDV**W**NSS**A**	0.3±0.34
4	FEDV**F**NSS**E**	28±1.23
5	FEDV**A**NSS**E**	48±1.45
6	FEDV**F**NSS**A**	>100
7	FEDV**A**NSS**Y**	>100
8	FEDV**A**NSS**A**	>100

Concentrations of soluble ECD1-nuc constructs required for 50% inhibition of antibody binding to coated wild type ECD1-nuc are shown.

**Figure 1:**
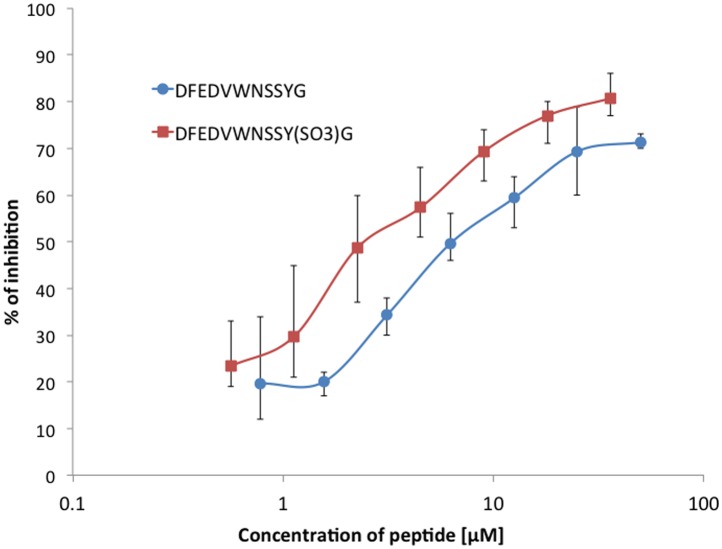
Inhibition of 2C3 MAb binding to ECD1-nuc. The antibody was incubated with serially diluted DFEDVWNSSYG (circles) or DFEDVWNSSY(SO_3_H)G (squares) peptides. Subsequent binding to ECD1-nuc was evaluated by ELISA as described in Materials and Methods. The difference in % of inhibition was statistically significant in the range of concentrations 3-60 nM (*P* < 0.05, two-tailed Mann-Whitney U test).

The influence of Tyr-30 sulfation was tested using sulfated and non-sulfated synthetic peptides encompassing amino acid residues 21-31 of ECD1 and evaluating ability to inhibit binding to ECD1-Nuc: results of the inhibition of 2C3 MAb binding to ECD1-Nuc by these peptides evaluated by ELISA is shown in Fig. 1. The SPR analysis revealed that concentration required for 50% inhibition of 2C3 MAb binding is 410 nM (±40) for DFEDVWNSSYG and 252 nM (± 20) for DFEDVWNSSY(SO_3_)G. Thus, sulfation of Tyr-30 is associated with a higher binding ability of the peptide to the antibody. In addition, to further investigate the role of sulfation of Tyr-30, we modified this residue in ECD1-nuc immobilized on a Biacore chip. Because a sulfotransferase is not commercially available, we used Src kinase to introduce a phosphate to the Tyr-30 residue to mimic a sulfate group. As a control, we used an anti-phosphotyrosine antibody which bound to ECD1 only after Src kinase treatment (data not shown). The kinetic data showed that phosphorylation of Tyr-30 caused an increase of 2C3 affinity, mainly by increasing k_a_ (Table 2 rows 1 and 4).

**Table 2:** Kinetic constants of 2C3 Fab binding to ECD1-nuc and purified DARC measured by SPR.

	**Ligand**	**ka (M^-1^s^-1^)**	**kd(s^-1^)**	**K_D_(M)**
1	ECD1-nuc	5.39 × 10^4^	7.66 × 10^-4^	1.42 × 10^-8^
2	DARC from K562 cells	2.076 × 10^6^	5.472 × 10^-4^	2.626 × 10^-10^
3	DARC from K562 cells after deglycosylation	9.754 × 10^6^	2.757 × 10^-4^	2.826 × 10^-11^
4	ECD1-nuc after phosphorylation	1.692 × 10^6^	7.769 × 10^-4^	4.592 × 10^-10^

This set of experiments was completed with the studies performed using transfected K562 cells expressing wild type or mutated DARC. The binding of 2C3 antibody was evaluated by flow cytometry, and the strength of binding was estimated as a ratio between the percentages of cells bound by 2C3 MAb to the percentage of cells bound by anti-Fy^a^ antibody (clone 655), which recognizes the ^41^YGANLE^46^ epitope [25]. We found that only the cells transfected with constructs encoding Trp at position 26 were bound by the antibody with high affinity (Table 3 rows 1-5), whereas substitution of that residue with alanine resulted in a dramatic decrease of the binding (Table 3, rows 6-8). When Trp-26 was substituted with Phe, the binding was reduced five times, but was not completely abolished (Table 3, row 9), suggesting that an aromatic ring at position 26 may to some extent substitute for the indole present in Trp residues. The flow cytometry experiments also confirmed the role of Tyr-30 and tyrosine sulfation, since substitution of Tyr-30 by Ala caused a decrease of 2C3 MAb binding (Table 3, row 2), while substitution of Tyr-30 by Glu increased the binding to some extent (Table 3, row 3). These data were consistent in ELISA and SPR experiments.

**Table 3:** Binding of anti-Fy^a^ and 2C3 MAb to DARC mutants expressed in K562 cells measured by flow cytometry.

	**SEQUENCE**	**% pos 2C3 Mab % pos Fy^a^ MAb**
1	FEDV**W**NS**SY**	0.97±0.07
2	FEDV**W**NS**SA**	0.75±0.03
3	FEDV**W**NS**SE**	1.16±0.09
4	FEDV**W**NSAY*	0.97±0.04
5	FEDV**W**NSAA*	0.95±0.03
6	FEDV**A**NS**SY**	0.04±0.01
7	FEDV**A**NSAE*	0.08±0.02
8	FEDV**A**NSAY*	0.00
9	FEDV**F**NS**SY**	0.19±0.02

Ratio of percentage of cells recognized by 2C3 antibody to percentage of cells recognized by anti-Fy^a^ antibody obtained for each clone is shown. Cells showing mean fluorescence above 10^2^ were considered as a positive.

* site N27 is not glycosylated

To evaluate the influence of glycosylation on 2C3 MAb binding, the recombinant DARC purified from K562 cells (S1 Fig.) was immobilized on the chip and the oligosaccharides were removed by PNG-ase F. The *Sambucus nigra* agglutinin (SNA) lectin which recognizes terminal residues of sialic acids was used to evaluate if the reaction was complete. It was shown before that terminal sialic acid residues are present in oligosaccharide chains of DARC [20, 21]. We found that binding of SNA was largely decreased after treatment of the immobilized protein by PNG-ase F (data not shown), while affinity of the antibody increased (Table 2, rows 2 and 3), suggesting that oligosaccharide chains, which are not part of the epitope, may in fact hinder partially access of the antibody to the peptide backbone.

In contrast, when binding of 2C3 MAb was evaluated using K562 cells transfected with the vector encoding DARC without glycosylation site at Asn-27, no difference in binding was observed between such construct and consensus DARC (Table 3, compare rows 1 and 4). It may be argued that the affinity increase observed in the SPR experiment could not be detected when the less sensitive flow cytometry technique was employed.

### Cloning and expression of 2C3 Fab

The 2C3 Fab fragment was cloned and expressed in *E. coli* and purified (S2 Fig.), and evaluated by ELISA and surface plasmon resonance (Table 4). The accession numbers for variable regions of the light and heavy chains are KP179400 and KP179401, respectively.

**Table 4:** Kinetic constants of 2C3 MAb and 2C3 Fab binding to ECD1-nuc measured by SPR.

	**k_a_**	**k_d_**	**K_D_**
2C3 Fab	0.998 × 10^4^	9.259 × 10^4^	9.305 × 10^-8^
2C3 MAb	4.62 × 10^4^	5.11 × 10^-5^	1.1 × 10^-9^

**Figure 2:**
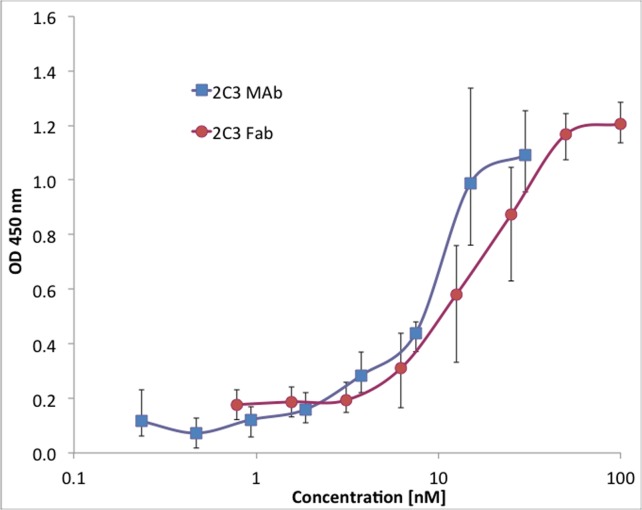
Comparison of binding of 2C3 MAb and 2C3 Fab to ECD1-nuc evaluated by ELISA. The binding of 2C3 MAb (squares) and 2C3 Fab (circles) was evaluated by ELISA as described in Materials and Methods. The difference in binding is statistically significant at concentration 20 nM (*P* < 0.05, two-tailed Mann-Whitney U test).

It was found that the affinity of 2C3 Fab is about two times lower (statistically significant at concentration 20 nM, *P* < 0.05, two-tailed Mann-Whitney U test) than the affinity of 2C3 parent antibody (Fig. 2).

The lower affinity is an asset to STD-NMR studies as discussed below.

### STD-NMR studies of interaction between 2C3 Fab and its epitope

**Figure 3:**
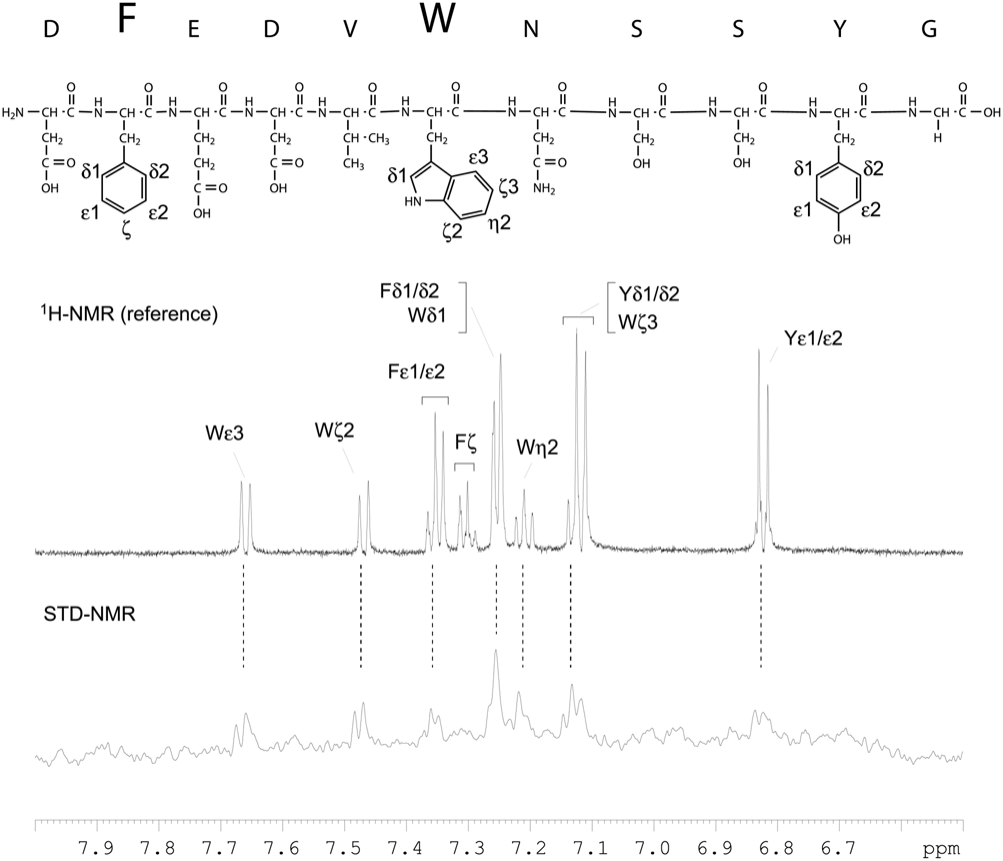
STD-NMR analysis of the Fab-binding epitope. The STD NMR (bottom) and reference ^1^H NMR spectra of the peptide DFEDVWNSSYG (diagram) in the presence of Fab were acquired at 600 MHz and 25°C for samples (160μl, in a 3 mm tube) prepared in PBS made with ^2^H_2_O, pH 7.5. The protein was irradiated at δ_H_ 9.5 ppm (on-resonance) and δ_H_30 ppm (off-resonance) with a saturation time of 2 s. The excitation sculpting pulse sequence was used to suppress water signals. The oversized one-letter amino acid codes indicate the proton resonances that were enhanced in the STD experiments. The Greek letters and numbers designate the enhanced resonances of the aromatic systems. The unresolved resonances are grouped.

The role of structural elements within the peptide that contribute to the Fab-binding epitope were investigated by STD NMR [34, 35] using synthetic peptide DFEDVWNSSYG. We found that the enhanced resonances in the STD NMR spectrum belonged predominantly to protons ε3, ζ2, ζ3, η2 and δ1 of the Trp-26 residue (the enhanced resonances are indicated in Fig. 3). Minor enhancements of the resonances of protons ε1/ε2 and δ1/δ2 of the Phe residue were also observed. The enhanced resonance at ~7.12 ppm could not be assigned unequivocally as protons δ1/δ2 of Tyr residue and proton ζ3 of the Trp were not resolved. Minute enhancements of Tyr protons ε1/ε2 were only noticeable when the on-resonance irradiation frequency was set to 9.5 ppm. Additionally, no substantial enhancements were observed in the region of signals from aliphatic side chains. Interestingly, no enhancements of valine (V) CH3 proton resonance were observed. No enhancement of Val-35 STD signal gives evidence of either the lack of binding or too strong binding and low off rate value or insufficient energy transfer from the protein to this residue due to the unfavorable conformation causing the distance too large for STD signal observation. The STD-NMR experiments confirmed that the immunodominant epitope recognized by the Fab is limited predominantly to the Trp residue within the peptide. However, the intensities of the enhanced signals observed in the STD NMR spectrum were low. This could be explained by the tight binding of the Fab to the peptide and presumably small off rates (k_off_) resulting in an inefficient saturation transfer to ligand.

### Modeling of the peptide structure

In order to figure out why Val-25, which seemed to be indispensable part of the eptiope, does not interact directly with the antibody, structures of peptide DFED**V**WNSSYG and peptides with Val-25 substituted with other amino acids were evaluated using PEP-FOLD webserver. The most relevant peptides conformations, *i.e*. associated with lowest sOPEP energies, were selected for further analysis (S1 Table). Based on a structural alphabet approach [36] and a coarse-grained protein force field for structure fold prediction [37], PEP-FOLD proposed various local peptide structures near to their native conformations. Experimental 3D structure of the Gln-19—Tyr-30 portion of DARC was extracted from published DARC-PvDBP complex (PDB 4NUU) [5], it was compared with peptide models obtained in our in silico studies. Structures of peptide models based on sOPEP energy show striking similarities to the experimental one. The degree of similarity of experimental and predicted structures was evaluated by computing backbone RMSD (root-mean square deviation): RMSD values range between 0.69 and 0.83 Å. This strongly supports the pertinence of our modeling approach. Moreover the distances between Trp-26, Tyr-30 and Phe-22 in the peptide DFED[V, I, E, L]WNSSYG, were calculated (S1 Table), and show some significant differences underlining the influence on the peptide structure of the amino acid present at position 25.

**Figure 4:**
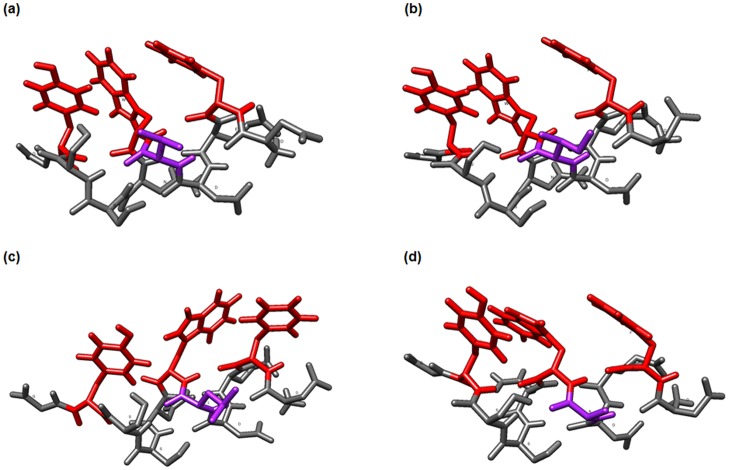
Three-dimensional structure of the peptide DFEDVWNSSYG (a) and peptides in which Val-25 is substituted by amino acids of different hydrophobicity (b-d). Peptide structures were calculated in PEP-FOLD and then visualized and analyzed in Chimera software. The amino acid residues involved in the antibody binding are visualized in red, while the residue that was changed: Val (a), Ile (b), Leu (c) and Ala (d) is visualized in purple.

Indeed the peptide models shown in Fig. 4 and S1 Table demonstrate how Val-25 substitution alter the epitope 3D structure and may influence binding to 2C3 MAb. The distances between residues involved in binding of the antibody are slightly different for those models. The substitution of Val (HI = 4.2) with Ile residue, an amino acid with higher hydrophobic index (HI = 4.5) but longer aliphatic chain does not cause significant structure deformation (0.45 to 0.66 Å more than for Val-25 model, S1 Table). Val-25 substitutions with amino acids such as Ala (HI = 1.8) and Leu (HI = 3.8) change the epitope conformation and extend the distances between amino acids involved in the interaction with antibody 2C3. Leu and Ala substitutions imply 0.09-2.019 and 0.15-2.29 Å shifts compared to Val-25 model, respectively.

Moreover, conformational changes of particular amino acids side-chains, which may impact the epitope formation, were also observed (Fig. 4).

### Discussion

As the role of monoclonal antibodies in biology and medicine becomes increasingly important, so does the precise evaluation of the epitopes being recognized by the antibodies. There are many methods allowing to precisely define amino acid residues that are involved in antibody binding, such as evaluation of binding of the antibody to synthetic peptides (Pepscan analysis) or to recombinant peptides expressed either in bacteria on in eukaryotic cells [26, 38–43]. Recently, saturation transfer difference spectroscopy NMR (STD-NMR) has been increasingly used in epitope-antibody studies, although majority of antibodies evaluated by this method bind carbohydrate antigens [44–46]. In this paper we used a variety of methods, including STD-NMR, to precisely define the peptidic epitope of 2C3 MAb.

The murine monoclonal antibody 2C3 is one of the widely used anti-DARC antibodies. Since its binding interferes with some of the DARC functions such as chemokine binding [24], and invasion of erythrocytes by *P. vivax* merozoites [27], precise knowledge on the recognized epitope seems necessary for its potential applications.

Our results unequivocally re-establish the important role of Trp-26 in DARC recognition by the antibody confirming the data published by Wasniowska [24], as it cannot be replaced by any residue without significant decrease of affinity. However, when Trp-26 is substituted by Phe, binding of 2C3 MAb was not abolished completely, suggesting that the aromatic ring may substitute the indole ring. The role of Tyr-30 has also been clarified: it can be substituted with Ala without strongly decreasing the affinity, which stands in contrast with the results published previously [25]. However, our results suggest that sulfation of Tyr-30 causes an increase of the antibody affinity to some extent. Moreover, when ECD1-nuc construct contained both Phe at position 26 and Glu at position 30, the binding of the antibody increased in comparison to the construct with Phe-26 only. Finally, phosphorylation of Tyr-30 on ECD1-nuc used to mimic sulfation, caused and increase of the 2C3 MAb affinity. These data suggest that the negative charge at Tyr-30 possibly increases the antibody—antigen interaction.

Results of STD-NMR presented in this paper support these conlusions. Saturation transfer difference (STD)-NMR technique can be used to identify the proton resonances of a ligand in close contact with the binding protein: side chains within the ligand that interact most strongly show a strong STD effect. There are several reports in which interaction between receptor and peptide ligand [47, 48] or monoclonal antibody and oligosaccharide epitope [44, 49, 50] is evaluated. However, only a handful of papers exist in which interactions between monoclonal antibody and peptide epitope are studied [45, 51]. The main limitation of the technique is the dissociation constant, which must be in range 10^-3^ to 10^-8^M [34, 35]. In the case of 2C3 antibody, the K_D_ is too low (1.1 × 10^-9^M) so we decided to use Fab fragment, whose K_D_ is 9.3 × 10^-8^M. Evaluation of interaction of peptide DFEDVWNSSYG with 2C3 Fab by STD-NMR technique confirmed that Trp-26 plays a major role in the antibody binding. It was found that interaction between Phe-22 and the antibody is weaker, whereas Tyr-30 shows the weakest interaction. Thus, only aromatic residues have been identified by STD-NMR, while other residues (such as Val-25) cannot be resolved in the spectrum. The Val-25 seems to play an important role in the epitope, because it was shown before that it cannot be replaced by another residue (with exception of Ile) without losing ability to bind 2C3 antibody [24].

In order to explain the possible role of Val-25 in the epitope, we used *in silico* modeling of DFEDVWNSSYG peptide, and of peptides with Val-25 substituted by Ile, Leu or Ala. Our models suggest that Val-25 substitution by Leu or Ala implies changes in peptide conformation and moves slightly the hydrophobic amino acids away from each other. However, it must be noted that the Ile side chain (which is longer than Val) might have an influence on epitope formation. The modeling suggests that Val-25 might cramp with other hydrophobic amino acids and control the polypeptide structure. Thus, substitution of Val-25 with other amino acid residues with different hydrophobicity may alter this structure by rendering Tyr-30 further from the epitope (S4B Fig.). Modeling studies showed that the essential residues forming the epitope (Phe-22, Trp-26 and Tyr-30) are exposed one face of the helix while Val-25 located on the opposite face of the helix might not participate to contacts with antibody. We hypothesize though that Ile, which has a longer side chain than Val, might in some way make some steric hindrance to the antibody epitope interaction since modeling suggests that it does not modify grossly epitope structure. In summary, the peptide structure of the epitope seems to be compact, with aromatic residues forming a single block on one side of an helix (Fig. 4).

Taken together, our results confirm that the minimal DARC epitope recognized by 2C3 MAb is linear and consists of five amino acids: ^22^FEDVW^26^, but there is a certain interaction between Tyr-30 and the antibody as shown by STD-NMR. In addition, we found that when Tyr-30 was substituted by a residue with a negative charge, such as glutamic acid, binding of 2C3 antibody was slightly increased despite other crucial amino acid substitutions, suggesting that the negative charge at amino acid 30 may play an auxiliary role in recognition. *In vivo*, the negative charge is provided by the Tyr-30 due to its sulfation.

Our results were obtained using a set of methods that are applicable for establishing epitope of any monoclonal antibody. We have demonstrated that using eukaryotic and prokaryotic expressed constructs, any doubts regarding antibody specificity can be clarified. We also showed usefulness of SPR analysis to demonstrate influence of post-translational modifications by carrying out enzymatic reactions “in situ” on the protein previously immobilized on the chip. In addition, we showed that STD-NMR technique can be used to evaluate binding of an antibody to a peptide, which to our knowledge is one of the first experiments of this kind described in literature.

## Supporting Information

S1 Fig Analysis of immunoaffinity chromatography on immobilized anti DARC VHH. Lanes 1: molecular weight standard (molecular weight of bands is noted on the left), supplemented with purifed anti DARC nanobody. Lane 2 and 4 20 μl of fractions collected during wash of the column with Tris saline 0.3% C12E8 buffer. Lane 3 and 5: 20 μl of material eluted from the coulmn with DFEDVW peptide. Lane 6 contains 20 μl of material eluted from column with 0.1 M glycine buffer. Lanes 1-3: silver nitrate staining, lanes 4-5: Western blotting with 2C3 MAb.
(TIF)

S2 Fig SDS-PAGE analysis of purified soluble 2C3 Fab. Lane 1: molecular weight standards. The purified protein (4 μg) were loaded onto 10% polyacrylamide gel in the absence (A) or presence (B) of 2-mercaptoethanol, and visualized with Coomasie Brilliant Blue.
(TIF)

S3 Fig Inhibion of binding of 2C3 MAb to ECD1-nuc constructs evaluated by ELISA. 2C3 MAb was incubated with ECD1-nuc constructs in different concentration as described in Materials and Methods, and subsequent binding to ECD1-nuc—covered plates was evaluated by ELISA.
(TIF)

S4 Fig The 3-D structure of DARC bound to PvDBP (PDB 4NUU) as visualized by Chimera software (A) and compared to peptide models obtained in *in silico* studies (B). DBP-RII molecules are shown in blue and green, DARC molecule is shown in red [5]. DARC was enlarged on the right and the crucial residues were highlighted using different colors: Phe-22 (purple), Val-25 (red), Trp-26 (green), Tyr-30 (blue). Q19-Y30 peptide from DARC (red) was enlarged and compared to peptide models (black) obtained in *in silico* studies: (a) DFEDVWNSSYG (RMSD = 0,75), (b) DFEDIWNSSYG (RMSD = 0,83), (c) DFEDLWNSSYG (RMSD = 0,70), and (d) DFEDAWNSSYG (RMSD = 0,67); B. 3-D structure of peptides obtained in modelling studies. Amino acids exposed on the same face of the helix, involved in antibody binding, are shown in purple (Phe-22), green (Trp-26) and blue (Tyr-30). Residues located on the other surface of the helix (Val-25, Ile-25, Leu-25 and Ala-25) are shown in red.
(TIF)

S1 Table The representative structure models of the native DFEDVWNSSYG peptide and peptides with Val-25 substitution. Three-dimensional structure of sOPEP energy model was calculated by PEP-FOLD server. The distances between Trp-26, Tyr-30 and Phe-22 were calculated by Chimera software. The colors show hydrophobicity of surface amino acids in the Kyte-Doolittle scale (blue for the most hydrophilic to white at 0.0 and to orange red for the most hydrophobic). Peptide models underline the influence of hydrophobic/hydrophilic amino acids on position 25 (Val, Ile, Leu, Ala) on peptide folding.
(DOCX)
