# Supplementary figures and images for: Studies of a Murine Monoclonal Antibody Directed against DARC: Reappraisal of Its Specificity
Source: PLoS One. 2015 Feb 23;10(2):e0116472. doi: 10.1371/journal.pone.0116472 (PMC4338028; doi:10.1371/journal.pone.0116472)

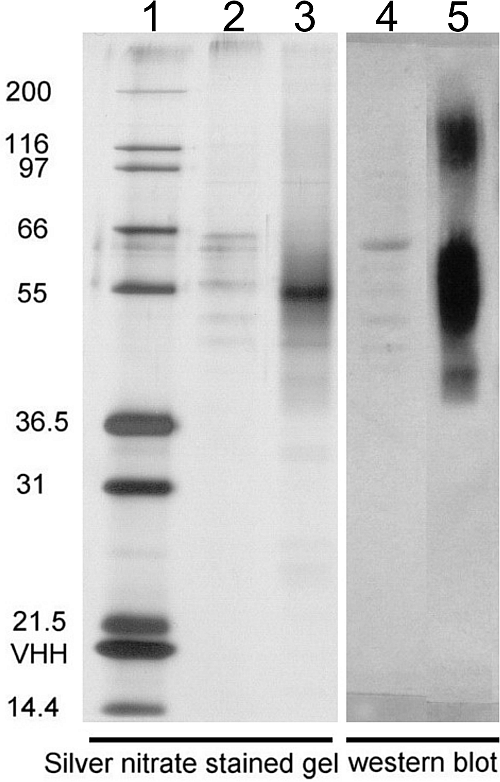

Supplement: S1 Fig — Lanes 1: molecular weight standard (molecular weight of bands is noted on the left), supplemented with purifed anti DARC nanobody. Lane 2 and 4 20 μl of fractions collected during wash of the column with Tris saline 0.3% C12E8 buffer. Lane 3 and 5: 20 μl of material eluted from the coulmn with DFEDVW peptide. Lane 6 contains 20 μl of material eluted from column with 0.1 M glycine buffer. Lanes 1-3: silver nitrate staining, lanes 4-5: Western blotting with 2C3 MAb. (TIF) [file pone.0116472.s001.tif]

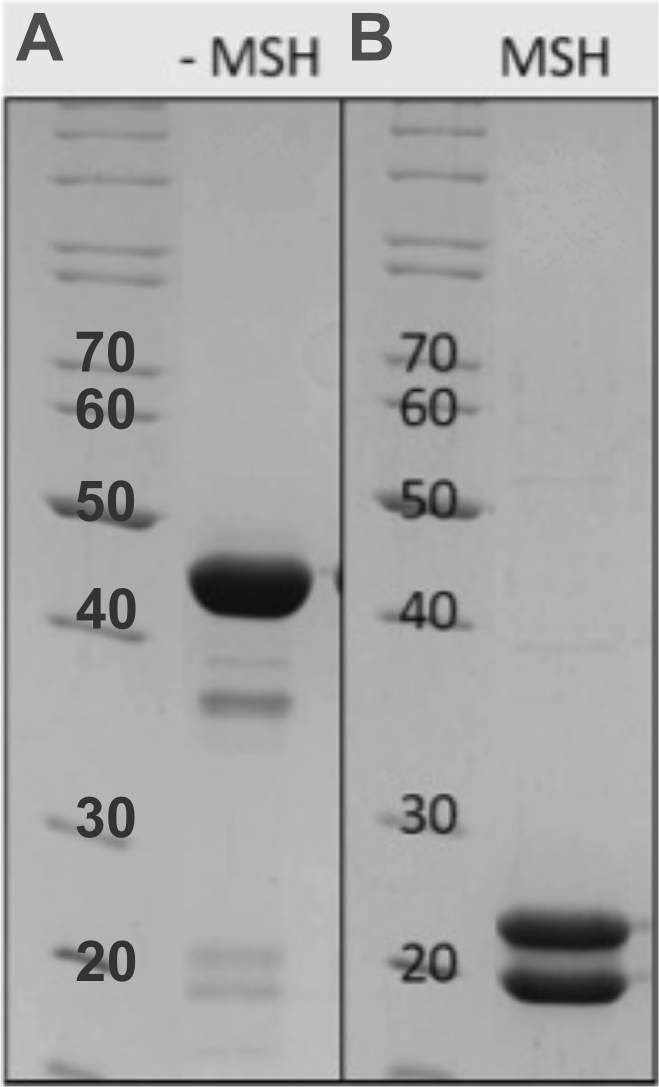

Supplement: S2 Fig — Lane 1: molecular weight standards. The purified protein (4 μg) were loaded onto 10% polyacrylamide gel in the absence (A) or presence (B) of 2-mercaptoethanol, and visualized with Coomasie Brilliant Blue. (TIF) [file pone.0116472.s002.tif]

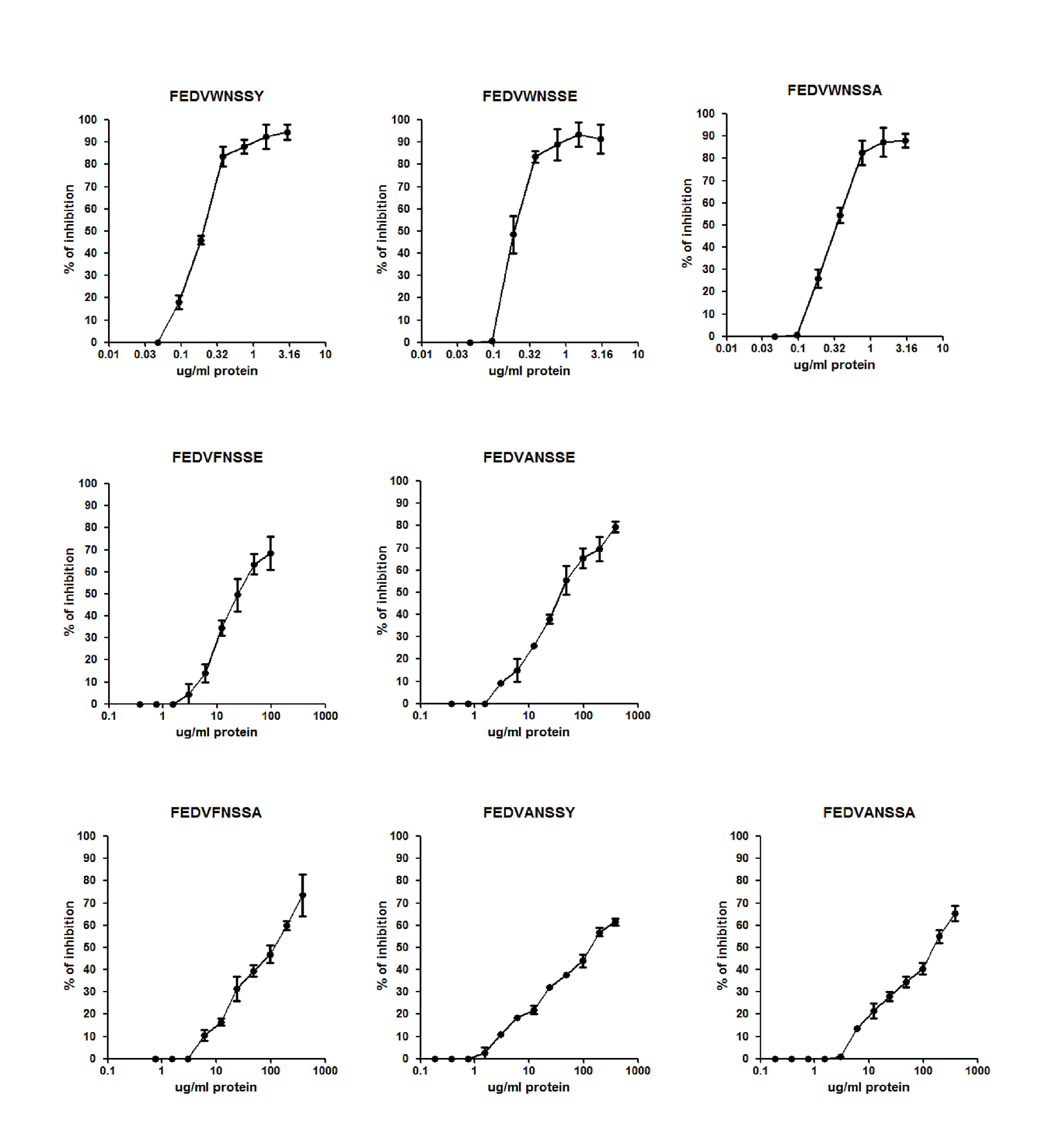

Supplement: S3 Fig — 2C3 MAb was incubated with ECD1-nuc constructs in different concentration as described in Materials and Methods, and subsequent binding to ECD1-nuc—covered plates was evaluated by ELISA. (TIF) [file pone.0116472.s003.tif]

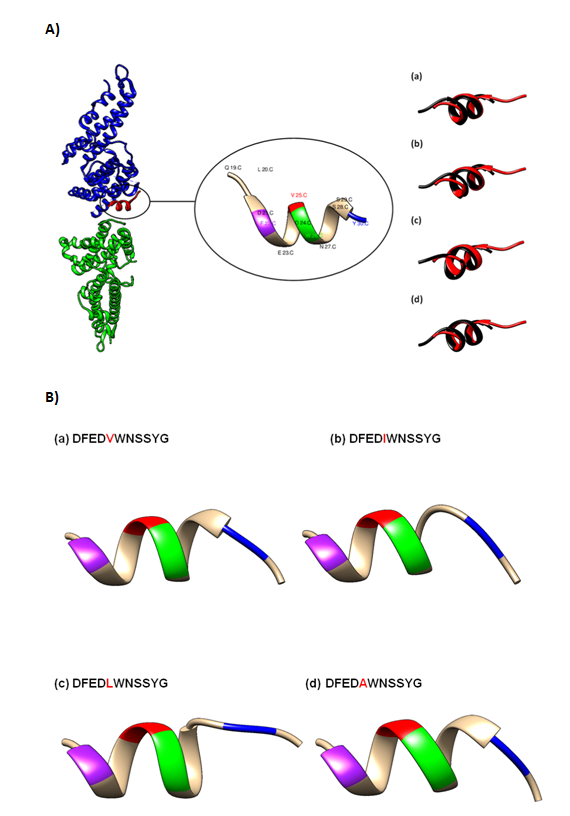

Supplement: S4 Fig — DBP-RII molecules are shown in blue and green, DARC molecule is shown in red [5]. DARC was enlarged on the right and the crucial residues were highlighted using different colors: Phe-22 (purple), Val-25 (red), Trp-26 (green), Tyr-30 (blue). Q19-Y30 peptide from DARC (red) was enlarged and compared to peptide models (black) obtained in in silico studies: (a) DFEDVWNSSYG (RMSD = 0,75), (b) DFEDIWNSSYG (RMSD = 0,83), (c) DFEDLWNSSYG (RMSD = 0,70), and (d) DFEDAWNSSYG (RMSD = 0,67); B. 3-D structure of peptides obtained in modelling studies. Amino acids exposed on the same face of the helix, involved in antibody binding, are shown in purple (Phe-22), green (Trp-26) and blue (Tyr-30). Residues located on the other surface of the helix (Val-25, Ile-25, Leu-25 and Ala-25) are shown in red. (TIF) [file pone.0116472.s004.tif]
