# Supplementary material for: Studies of a Murine Monoclonal Antibody Directed against DARC: Reappraisal of Its Specificity
Source: PLoS One. 2015 Feb 23;10(2):e0116472. doi: 10.1371/journal.pone.0116472 (PMC4338028; doi:10.1371/journal.pone.0116472)
Supplement: S1 Table — Three-dimensional structure of sOPEP energy model was calculated by PEP-FOLD server. The distances between Trp-26, Tyr-30 and Phe-22 were calculated by Chimera software. The colors show hydrophobicity of surface amino acids in the Kyte-Doolittle scale (blue for the most hydrophilic to white at 0.0 and to orange red for the most hydrophobic). Peptide models underline the influence of hydrophobic/hydrophilic amino acids on position 25 (Val, Ile, Leu, Ala) on peptide folding. (DOCX) [file pone.0116472.s005.docx]

| Peptide | HI* | sOPEP | Distance | | | 3D structure |
| --- | --- | --- | --- | --- | --- | --- |
|  |  |  | F-W [Å] | W-Y [Å] | Y-F [Å] |  |
| DFED**V**WNSSYG | 4.2 | -10.52 | 6.343 | 4.935 | 9.835 | **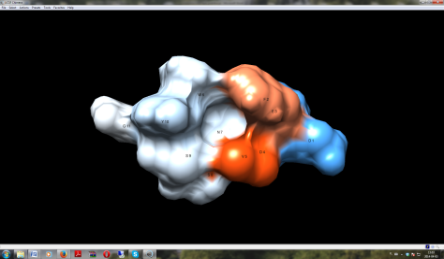** |
| DFED**I**WNSSYG | 4.5 | -10.30 | 6.907 | 5.381 | 10.491 | **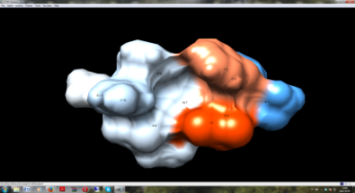** |
| DFED**L**WNSSYG | 3.8 | -12.49 | 6.253 | 6.360 | 11.854 | **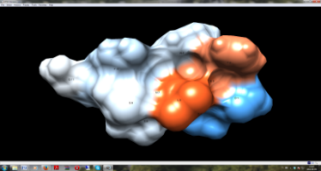** |
| DFED**A**WNSSYG | 1.8 | -11.18 | 6.189 | 6.056 | 12.130 | **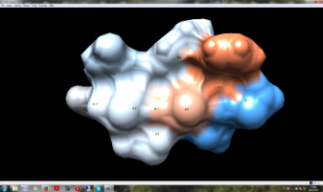** |

* Hydrophobicity index for amino acid on 5^th^ position (Kyte-Doolittle scale)
